# The Role of Nano-TiO_2_ Lubricating Fluid on the Hot Rolled Surface and Metallographic Structure of SS41 Steel

**DOI:** 10.3390/nano8020111

**Published:** 2018-02-16

**Authors:** Yanan Meng, Jianlin Sun, Ping Wu, Chang Dong, Xudong Yan

**Affiliations:** 1 School of Materials Science and Engineering, University of Science and Technology Beijing, Beijing 100083, China; mynfighting@163.com (Y.M.); ldtwuping@buu.edu.cn (P.W.); ustb_dc@163.com (C.D.); bachelordong5969@163.com (X.Y.); 2 Departments of Foundational Science, Beijing Union University, Beijing 100101, China

**Keywords:** nano-lubrication, tribology, hot rolling, surface topography

## Abstract

In this paper, nano-TiO_2_lubricating fluid was chosen as an advanced rolling lubricant to investigate its effect on the hot rolled surface and metallographic structure of SS41 steel strips. The tribological performances of nano-TiO_2_ lubricating fluid were measured by a four-ball tribotester. The hot rolling experiments under different lubrication conditions were carried out by a four-high rolling mill. The surface morphology, oxide scales and metallographic structure after hot rolling were observed using a confocal laser scanning microscope and scanning electron microscope (SEM), respectively. The composition of surface attachments was analyzed with X-ray photoelectron spectroscopy (XPS). The results indicate that the nano-TiO_2_ lubricating fluid has a better tribological performance. The surface defects on the hot rolled surface could be decreased. The phase composition of the surface still appears as a mixture of ferrite and pearlite. The surface of steel strips is not micro-alloyed with titanium as predicted. Additionally, the grain size of rolled steel strips which were lubricated with the nano-TiO_2_lubricating fluid decreased by nearly 50%, compared with traditional lubricating fluid. Furthermore, it was found that the thickness of the oxide layers on the surface reduced, whilst the Rockwell hardness of the oxide layers was enhanced as nano-TiO_2_ lubricating fluid was applied.

## 1. Introduction

Due to their special physical and chemical properties [1], such as excellent diffusivity, chemical activity, and the ability to self-repair and film-form [2], nanoparticles have extensive applications in lubrication. Nanoparticles, such as molybdenum disulfide (MoS_2_) [3,4], silicon dioxide (SiO_2_) [3,5], titanium dioxide (TiO_2_) [6,7,8] and aluminum oxide (Al_2_O_3_) [8], can create the appropriate conditions for machining process [9]. Recent studies have confirmed that nanoparticles have potential applications in the steel rolling process [10,11], machine cutting process [12,13,14], micro-drilling process [15], etc. Previous studies reported that nano-TiO_2_ has good friction-reducing, anti-wear, and cooling properties, so it can reduce the coefficient of friction and rolling force during the hot rolling process [16,17].

Although previous studies have verified that using nano-TiO_2_ as an additive can improve the tribological properties of lubricants, few researchers have considered the impact of heating effect during the hot rolling process. Existing studies have principally focused on the effectiveness of nano-TiO_2_ on the rolling force, friction coefficient and other macro-performances. In this paper, the role of nano-TiO_2_ lubricating fluid on the microstructure of hot-rolled metal surface was investigated with interest. As the size decreases, anatase titanium dioxide can attain a more stable shape than rutile titanium dioxide at room temperature [18] and hence, TiO_2_ nanoparticles used in nano-lubricating fluid are always in the anatase phase. The anatase phase is known to be metastable, while rutile is the stable phase. Therefore, the anatase phase will transform to the rutile phase under heat treatment at temperatures typically ranging between 600–900°C [19,20]. The fundamental structures of anatase phase and rutile phase nano-TiO_2_ are both octahedron structures consisting of titanium and oxygen elements. However, the fundamental structures of anatase nano-TiO_2_ are connected with the communal vertexes, while the fundamental structures of the rutile phase share the edges. Actually, there are some previous chemical bonds broken and some emerging chemical bonds formed during the heat treatment [21]. Therefore, the TiO_2_ nanoparticles used in nano-TiO_2_ lubricating fluid might have a chance to react with the surface steel during the hot rolling process and cause an inhomogeneity of the strips in structure and texture. The structural and textural inhomogeneity of steel strips would impact the processing properties and service performance of products [22].

In this paper, nano-TiO_2_ lubricating fluid was prepared, and its lubrication performance was evaluated, using a four-ball tribotester. The surface morphology, oxidation, metallographic structures and elemental compositions on the surface of the SS41 steel strips, lubricated with or without nano-TiO_2_ lubricating fluid, were compared, to explore these problems. This exploration will play an important role in the application prospect of nano-TiO_2_ lubricating fluid used in hot rolling.

## 2. Experimental

### 2.1. Materials

Anatase nano-TiO_2_ (TiO_2_ content, >95 wt %) with a nominal diameter of 90 nm was used as the lubricant additive in this study. To prepare uniform and stable nano-fluid, the nano-TiO_2_ cannot be solely added into deionized water because of the polymerization of nanoparticles [23]. Both the physical dispersion method and the chemical dispersion method were used to solve this problem. A magnetic stirrer and ultrasonic dispersion instrument were used for physical dispersion. Some water-soluble dispersants, such as sodium hexametaphosphate (SHMP) and sodium dodecyl benzene sulfonate (SDBS) have both hydrophilic groups and lipophilic groups. So, they were added for chemical dispersion. The nano-TiO_2_ lubricating fluid was prepared according to the formula shown in Table 1. This kind of liquid can stay stable for more than 7 days. Furthermore, traditional lubricating fluid containing every additive in Table 1, except anatase nano-TiO_2,_ was obtained as a control group. In this work, the traditional lubricating fluid was used to determine the interference of the other additives on the results.

As the four-high rolling mill used in hot rolling test was an experimental mill with a working roller dimension of Ф170 × 350 mm, 70 mm was chosen as the breadth of the initial materials to reduce the rolling force. In addition, the steel strips (initial dimensions, 200 × 70 × 30 mm^3^) used in the hot rolling test were provided by Jiuquan Iron & Steel (Group) Co., Ltd. The chemical compositions of the steel strips are listed in Table 2.

### 2.2. Tribological Test

**Figure 1:**
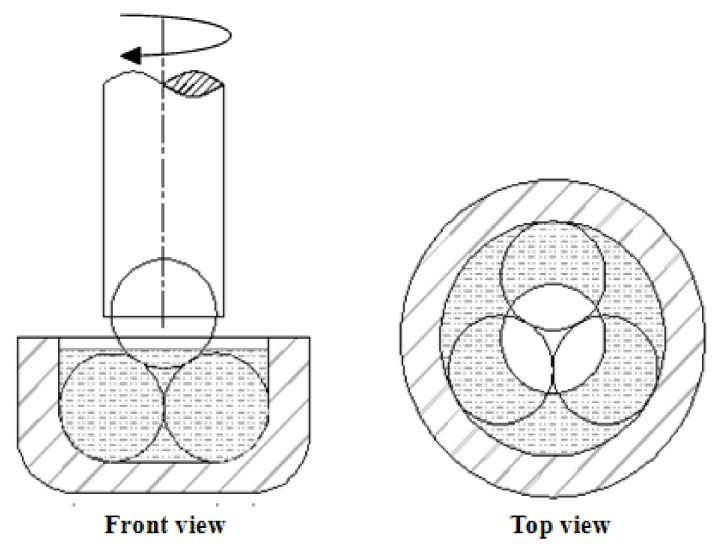
Schematic of the four-ball rolling friction.

The tribological tests were taken to represent the tribological properties of nano-TiO_2_ lubricating fluid. In addition, the same tests were conducted on the traditional lubricating fluid as a control trial. The maximum non-seizure load (P_B_), friction coefficient and wear scar diameter were measured on an MR-S10A four-ball tribometer, equipped with a rolling friction pair. The schematic of the four-ball rolling friction is shown in Figure 1.

A rotation velocity of 1760 r/min of the upper steel ball was applied in the representation of P_B_. While in the wear tests, the rotation velocity of the upper steel ball was 1200 r/min, the constant load was 392 N and the testing time was 30 min. The friction coefficients during the wear tests and the wear scar diameters after the wear test were measured to verify the friction reduction and antiwear behavior of nano-TiO_2_ lubricating fluid.

### 2.3. Hot Rolling Test

**Figure 2:**
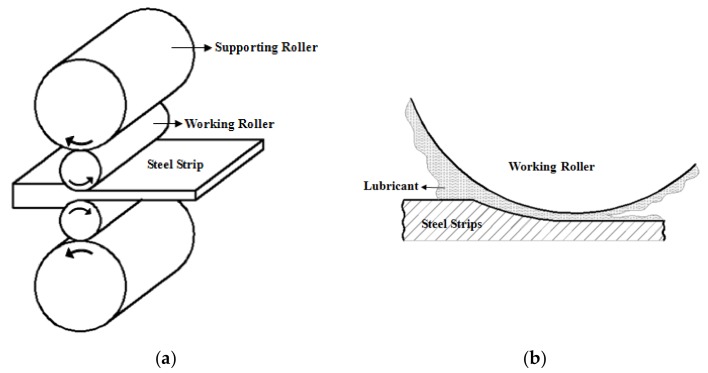
Sketches of (**a**) hot rolling and (**b**) lubrication during hot rolling.

The austenite phase has lower strength and hardness than the ferrite phase [24]—that is to say, the austenite phase possesses a preferable plasticity. To enhance the rolling efficiency, the hot rolling process should be taken completely in the austenitic phase. SS41 steel is a typical hypoeutectoid steel, wherefore the metallographic structure of SS41 steel is composed of a single austenite phase when the steels are heated above 800 °C. The strips were heated to 1150 °C, which is an empirical value, and held for 2 h to guarantee the temperature of the steel strips was above 850 °C during hot rolling. The nominal rolling speed was about 2.0 m/s, and the rolling pass was 5. The steel strips were hot rolled to 4 mm thick from 30 mm on the 4-high mill, and the corresponding rolling reductions were 26.7%, 27.3%, 37.5%, 40.0% and 33.3%, respectively. The sketch of hot rolling is shown in Figure 2a. Three kinds of lubricant conditions, namely, non-lubricant, traditional lubricating fluid, and nano-TiO_2_ lubricating fluid were used on the upper working roller in three-group comparative experiments, as shown in Figure 2b.

### 2.4. Characterization Methods

Four hot-rolled steel samples for each experimental group were prepared for analysis of surface morphology, oxidation, metallographic structure and surface chemical composition. The hot-rolled steel strips were cut into blocks of 10 × 10 mm from the center of the strips along the planes of the rolling direction (RD) and the normal direction (ND). The distance between each block was 20 mm.

The rolled surface of the first samples can be directly used for surface morphology and roughness observations after rinsing with petroleum ether. To provide an intuitive description of the surface quality of the hot-rolled steel strips, the surface morphology after hot rolling was characterized by a confocal laser scanning microscope (Olympus LEXTOLS 4000, Olympus, Japan) for 2D and 3D topography observation. The linear roughness perpendicular to the rolling direction was measured. To ensure the accuracy of the data, each group of the samples was measured five times and the determined value was obtained by averaging five measurements. To distinguish the form of wear, SEM (ZEISS EVO 18, Germany) was used for finer observation, and energy dispersive spectrometry (EDS, ZEISS, Germany) with a scanning electron microscope was applied to research the primary elementary composition of different forms of wear on the hot rolled surface.

The second samples used for oxidation investigation had to be milled and polished to Ra < 0.1 μm. To explore thickness of the oxide layers, the cross-sectional blocks were ground with SiC paper of 240, 400, 800, 1500 and 2000 mesh, orderly, and then polished by nylon fabric with a 2.5 mm diamond polishing paste. The oxide layers were observed by SEM with backscattered electron microscopy (BSE) and the thickness of the oxide layers were measured by EDS with elemental linear scanning. In this work, the elemental linear scanning was taken on the cross-section, with the scanning direction perpendicular to the rolled surface. The higher oxygen content represented the behavior of the oxide layers, while the lower oxygen content represented the simple metal. The Rockwell hardness of the oxide scales was conducted on a hardness tester; the applied load and holding time were 100 kg and 60 s, respectively.

The third samples were employed for the characterization of metallographic structure. The rolled surface of the samples had to be milled with SiC paper of 150 mesh to dislodge the oxide layers, and then milled and polished to Ra < 0.1 μm, using the same method mentioned in the last paragraph. After polishing, the surface was etched for 3–5 s with nitric acid alcohol, which was a mixture of 96% alcohol and 4% nitric acid, before microstructural analysis. The etched surface was surveyed by SEM, and the software of image analysis (Image Tool) was used to measure the grain size. To obtain a veracious datum, 12 adjoining grains in the image were chosen to be measured, and the average size was calculated, according to the 12 measurements. Then, the thicknesses of the samples were measured by micrometer and each final result was obtained by averaging six measurements.

The last samples were cut into thin plates, each with a thickness of 1.5 mm, for the X-ray photoelectron spectroscopy (XPS, Kratos, Japan), to determine the chemical states of typical elements on the strips’ surfaces.

## 3. Results and Discussion

### 3.1. Tribology Performance

The results of the tribology tests are shown in Table 3. The maximum non-seizure loads were measured according to GB/T 12583-1998. It can be seen in Table 3 that the maximum non-seizure load (P_B_) was signally enhanced by using TiO_2_ nanoparticles because of their remarkable film-forming property. This means the nano-TiO_2_ lubricating fluid can take effect under a greater positive pressure. The wear tests of each lubricant developed for 30 min, and the friction coefficients were measured and collected per second. The average friction coefficients of two lubricants were calculated to evaluate the friction reducing effect of using nano-TiO_2_. Wear scar diameters of the lower three steel balls were measured by optical microscope, and the mean values were calculated to evaluate the antiwear behavior of the nano-TiO_2_ lubricating fluid.

**Figure 3:**
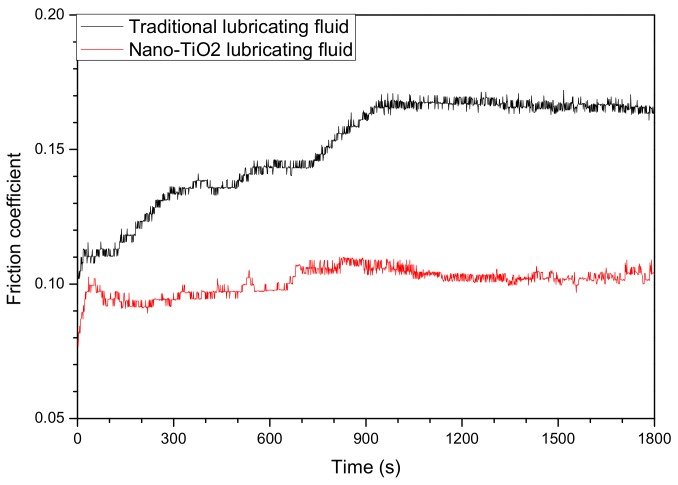
Friction coefficient-time curves of traditional lubricating fluid and nano-TiO_2_ fluid.

The friction coefficient-time curves of the traditional lubricating fluid and nano-TiO_2_ fluid of the upper steel ball under 392 N at 1200 r/min are shown in Figure 3. The friction coefficient of the traditional lubricating fluid presented a remarkably increasing trend with a prolonged time before 900 s and then became stable at about 0.17. As for the nano-TiO_2_ lubricating fluid, the friction coefficient was relatively consistent with the friction time. The results can exactly confirm the excellent film-forming property of nano-TiO_2_. Nano-TiO_2_ can easily adsorb to the steel surface to form a lubricating film. Thus, the friction coefficient-time curve of the nano-TiO_2_ lubricating fluid should be more stable.

### 3.2. Surface Morphology

**Figure 4:**
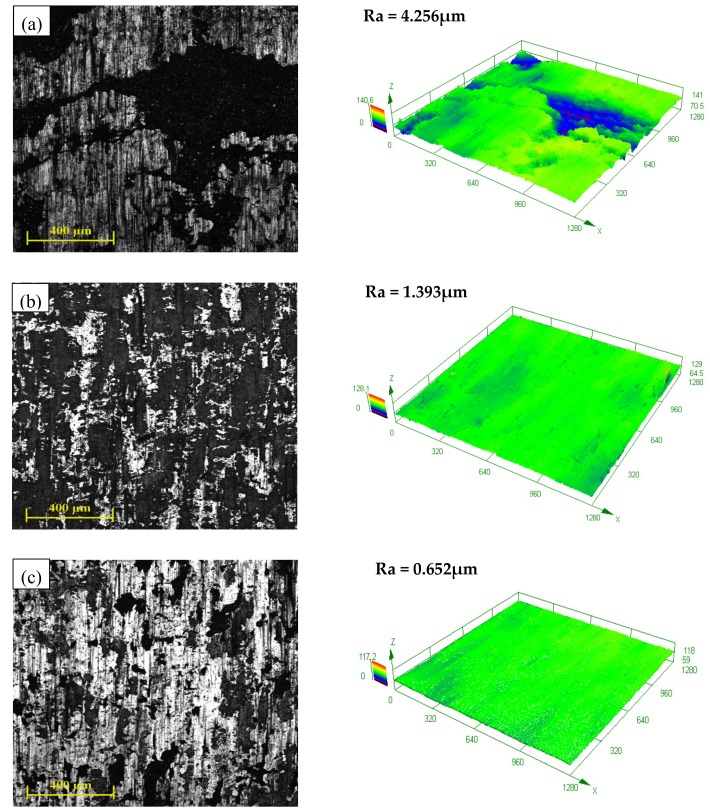
Optical micrograph and 3D topography of hot-rolled surface lubricated with (**a**) non-lubricant; (**b**) traditional lubricating fluid and (**c**) nano-TiO_2_lubricating fluid.

The optical micrograph and three-dimensional (3D) topography of hot-rolled strips under three different lubrication states were shown in Figure 4a–c . In Figure 4, a comprehensive representation was obtained from the optical micrograph. A surface micrograph of the hot-rolled strip shows signs of larger dark regions and dense scratches as no lubricant was used. There are also many wide and deep furrows that are perpendicular to the rolling direction on the non-lubricated surface. As a traditional lubricating fluid was used, there are very few transversal furrows that can be seen in the micrograph; furthermore, the size of dark regions and the deepness of dense scratches are both abated. However, many areas that are neither dark, nor bright, are exposed on the surface lubricated with traditional lubricating fluid. These areas might be due to the oxide wear that will be discussed in the following part. All these surface defects of the hot-rolled surface could be significantly reduced when the nano-TiO_2_ fluid was employed. It is known that optical microscopy has an inherently small depth field, whence the dark and bright regions can be associated with height difference. It indicates that the surface roughness of the hot-rolled strips would be decreased by using nano-TiO_2_lubricating fluid. These conclusions are confirmed by homologous 3D topographies in Figure 4. The data in the top left corner of the 3D topographies are the linear roughnesses of samples, which correspond to the change in rule of the surface topographies.

**Figure 5:**
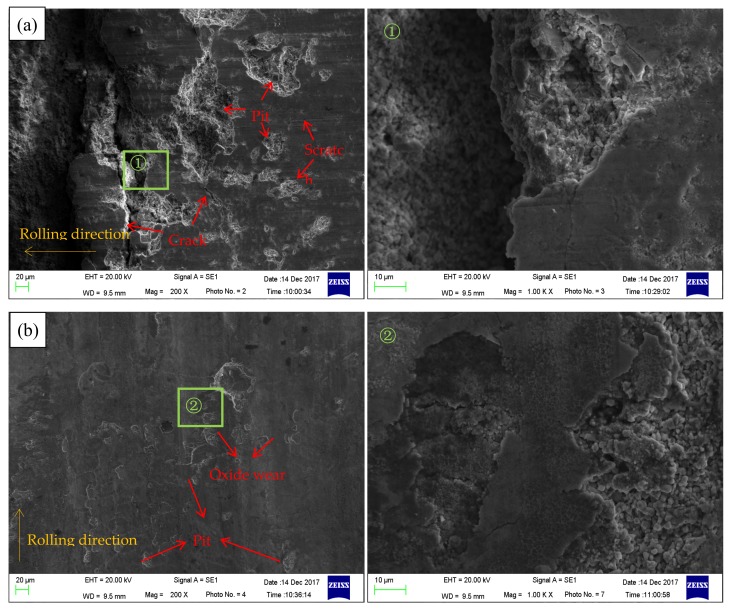

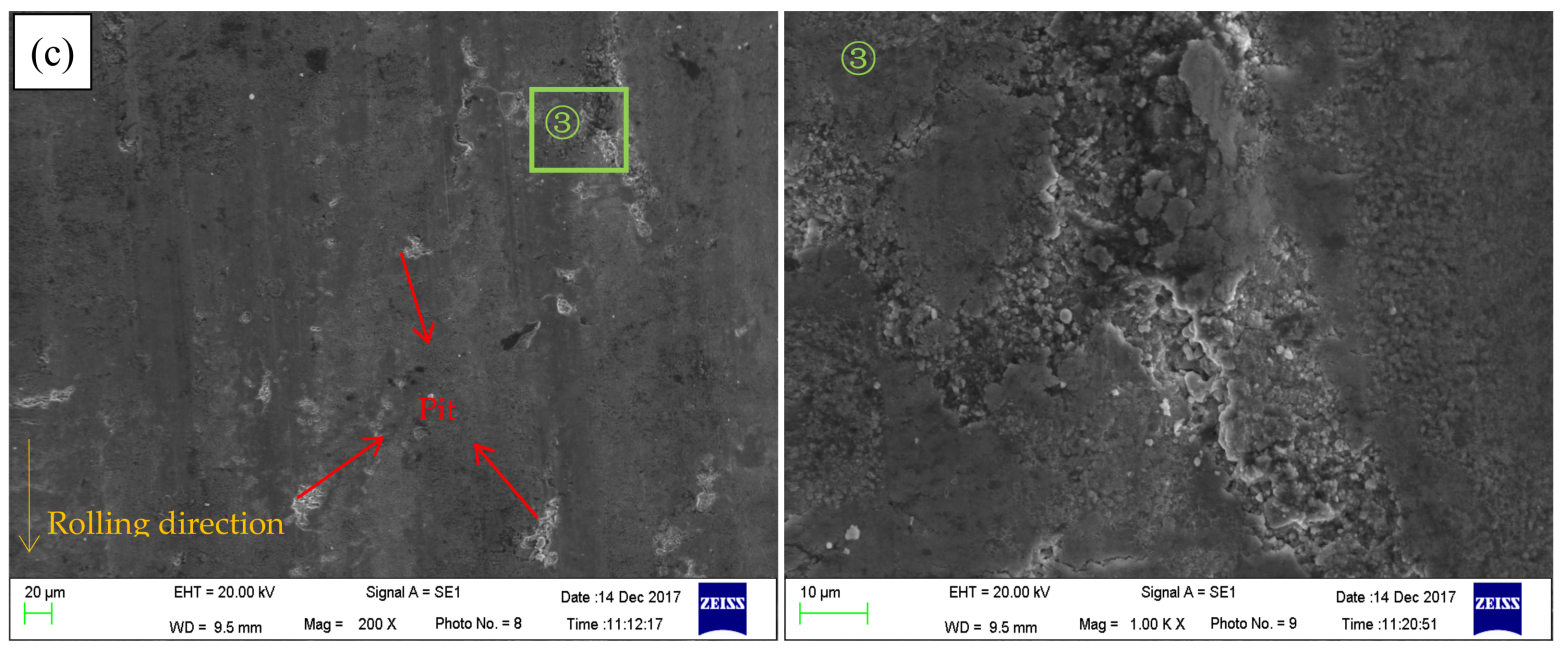
SEM images of hot-rolled surfaces lubricated with (**a**) non-lubricant; (**b**) traditional lubricating fluid and (**c**) nano-TiO_2_ fluid.

The SEM was used to distinguish and analyze these surface defects. The SEM images are shown in Figure 5. The irregular pieces of dark regions are metals desquamated from the strip surface because of the adhesive wear between the friction pairs. These metal fragments may come off the surface on which they are formed and be transferred back to the original surface [25]. As a result, the area whose surface steel avulsed from the strips is lower (appears blue in the 3D topographies), while the area that adhered to the metal fragments is higher (appears yellow in the 3D topographies). The adhesive wear of the strip surface will markedly influence the surface quality and strip yield. The pits pinpointed in Figure 5 can coincidentally correspond to this adhesive wear. The adhesive wear was verified to be reduced by using nano-TiO_2_ fluid, according to these results. Due to the adhesive wear, there must be some oxide scales and other hard materials adhered to the rolls. Therefore, the appearance of dense scratches parallel to the rolling direction is on account of scratching of the oxide scales and other hard materials adhered to the rolls. Comparing the size and depth of pits on three different surfaces, it is obvious that the pits on the non-lubricated surface are the largest, the pits in the traditional fluid lubricated surface take second place, and the pits in the nano-TiO_2_ fluid lubricated surface are smallest and shallowest. As a consequence, the scratches in Figure 5a are wider and deeper, while the scratches in Figure 5b,c are almost invisible. Moreover, the furrows perpendicular to the rolling direction must be cracks formed by the rupture of the steel surface. A crack might be a fearful surface defect which has a serious influence on the usability of the steel strips.

**Figure 6:**
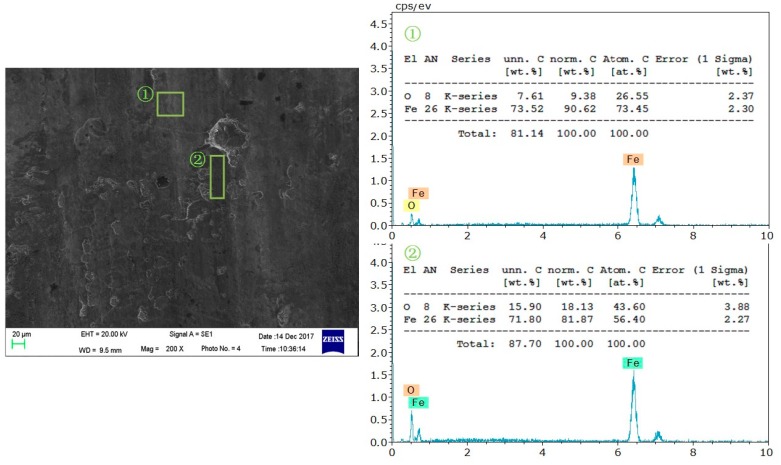
Energy dispersive spectrometry (EDS) spectra of strip surface lubricated with traditional lubricating fluid.

As shown in Figure 4b and Figure 5b, there are many regions with distinct colors on the surface lubricated with traditional lubricating fluid. These regions are speculated to be due to oxide wear as the traditional lubricating fluid was composed mainly of water. This inference was confirmed by EDS spectra of the strip surface lubricated with the traditional lubricating fluid, in Figure 6. The oxygen content of the abnormal region is higher than the normal region. This result indicates that there were some newly generated iron oxides in the abnormal region, which verified the previous assumption.

### 3.3. Oxide Scales

**Figure 7:**
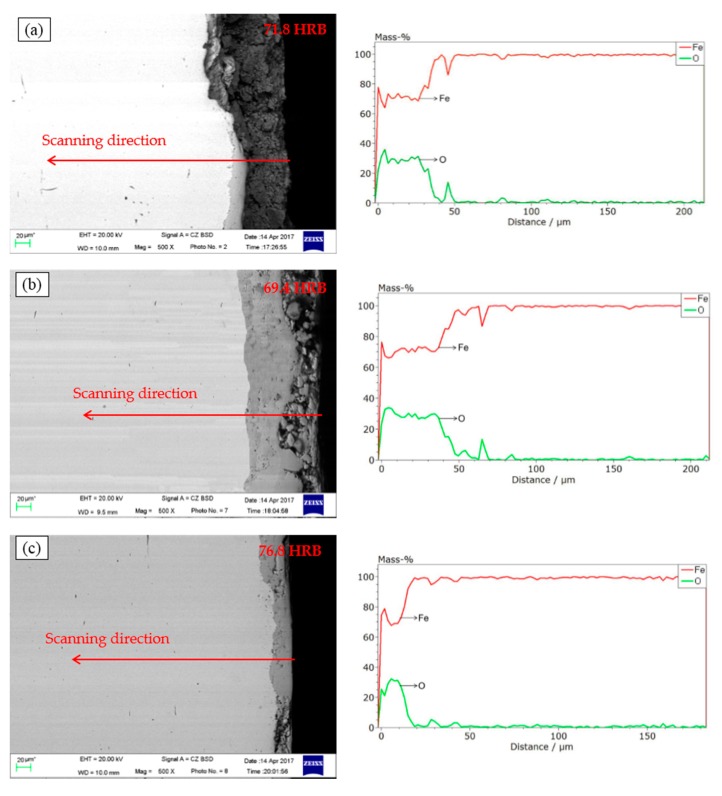
SEM images and EDS spectra of oxide scales of hot-rolled strips lubricated with (**a**) non-lubricant; (**b**) traditional lubricating fluid and (**c**) nano-TiO_2_ fluid.

Figure 7 illustrates oxide scales of hot-rolled strips under different lubricating conditions. The oxide scales of steel strips rolling without any lubricant are extremely loosened, as shown in the SEM image. As for the steel strips lubricated with the traditional lubricating fluid, this can hardly be improved. When nano-TiO_2_ fluid was used, the oxide scales become compact and fewer voids can be seen in the SEM image. The Rockwell hardness’ of the oxide scales listed in the top right corner of the SEM images are consistent with the visual results—the more compact the oxide scales are, higher the hardness is. The EDS with elemental linear scanning was taken to measure the thickness of the oxide scales, and the scanning direction is annotated on the SEM images. The vertical distributions of element Fe and element O in the transverse section were quantitated, and the spots whose oxygen content was above zero were considered to be the oxide scales. The thickness of the oxide layers without any lubrication was about 40 μm (Figure 7a). The thickness was increased to 60 μm when the traditional lubricating fluid was used (Figure 7b). In Figure 7c, the thickness decreased to 20 μm. The inferior antirust property of the traditional lubricating fluid was improved by using nano-TiO_2_.

**Figure 8:**
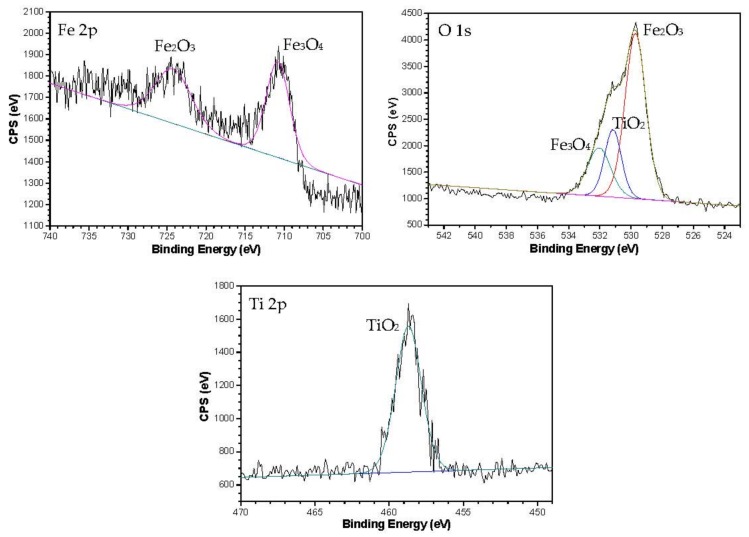
XPS spectra of Fe 2p, O 1s and Ti 2p on the oxide scales surface lubricated with nano-TiO_2_ fluid.

In order to explain the aforementioned phenomenon, XPS spectra were recorded to investigate the chemical states of the oxide scales. As shown in Figure 8, the Fe 2p peaks appeared at 710.80 eV and 724.22 eV. According to the NIST Standard Reference Data base 20 (version 4.1), the former, combined with the O 1s peaks at 529.72 eV, is assigned to Fe_3_O_4_, while the latter, combined with the O 1 s peak at 532.05 eV, is assigned to Fe_2_O_3_. The spectrum of Ti 2p shows a peak at the binding energy of 458.69 eV, combined with the binding energy of O1s 531.16 eV, which can be attributed to the chemical state of TiO_2_. The TiO_2_ signal indicates that nano-TiO_2_ has been deposited on the contact surfaces and forms a physical protecting film on the flat. In other words, the TiO_2_ nanoparticles might adsorb on the steel surface during the hot-rolling process, meanwhile, filling in the voids of the loosen oxide scales and isolating the air to prevent further rusting of the metallic matrix. Moreover, there is not any new phase formed on the steel surface—the nano-TiO_2_ could only create a physical adhesion film on the oxide scales to protect the steel.

### 3.4. Metallographic Structure

**Figure 9:**
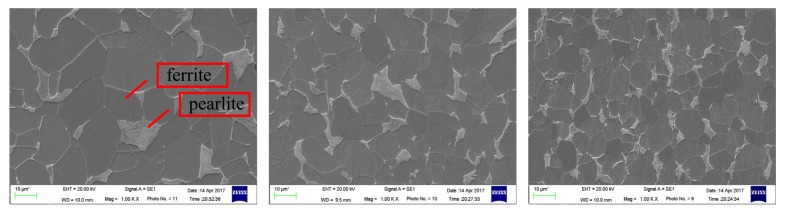
Microstructures of rolled strips for (**a**) non-lubricant; (**b**) traditional lubricating fluid and (**c**) nano-TiO_2_lubricating fluid.

As oxide scales of the third samples were dislodged, the metallographic structure of the metal base could be explored by SEM. The microstructures of the hot-rolled metal base after polishing and etching are shown in Figure 9. The metallographic structure of the metal base appears as a mixture of ferrite and pearlite, while the matrix phase is ferrite and the second phase in grain boundaries is pearlite. There was no novel phase formed when the nano-TiO_2_ was added. That means there could not have been any steel strip micro-alloyed with titanium. The reason for this inference will be explained in the following part. Furthermore, comparing Figure 9a–c , the grains are observed to be refined, obviously. The refined grains will lead to better mechanical properties and processing ability, with all else held equal [26].As the grains are subtler, the deformation can be easily coordinated—both the plasticity and tenacity can be improved. Additively, the flow stress of the steels would be enhanced; as a result, the materials could have a higher strength.

To make a quantitative comparison, the average sizes in the images were measured using the image analysis software (Image Tool); the results are shown in Table 4. The grain size of the rolled steel strips with the nano-TiO_2_ lubricant decreased by nearly 60% when compared with non-lubrication. In addition, the traditional lubricating fluid contributes about 25%.

The main reason for this phenomenon is the increase in rolling deformation. The finishing thicknesses and spring-back values of hot rolled steel strips were shown in Table 5. The rolling mill is known to have rebound resilience, so the actual thickness would thicker than the height of the roll gap, and the real thickness is the sum of the height of the roll gap and the spring-back value. The spring-back can be reduced by about 86.7% as the TiO_2_ nanoparticles are added into the lubricant, and the overall reduction in the same rolling period can be enhanced visibly. On account of the rise of rolling deformation, more and more dislocations and lattice defects appeared. As a result, the nucleation rate increased due to the enhancement of lattice distortion energy, and the grains became refined [27].

**Figure 10:**
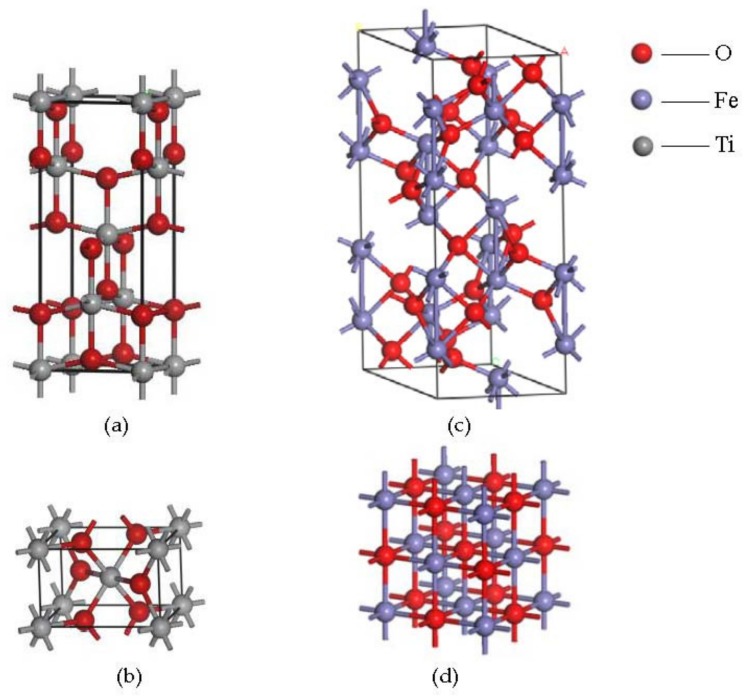
The crystal structures of (**a**) anatase nano-TiO_2_ (**b**) rutile nano-TiO_2_ (**c**) Fe_2_O_3_ and (**d**) FeO.

To explore the reason why no new phase appeared as the nano-TiO_2_ was added, the crystal structure of nano-TiO_2_ and oxide scales were analyzed. The element Fe on the surfaces exists in the forms, Fe^3+^ and Fe^2+^, and the titanium in nano-TiO_2_ is Ti^4+^. According to the crystal structures of TiO_2_, Fe_2_O_3_ and FeO (Figure 10), the ligancies of Ti^4+^, Fe^3+^ and Fe^2+^ are all six. This indicates that the replacement of these ions may occur, on account of the lattice imperfection. The incorporation of Ti^4+^ into the Fe_2_O_3_ crystal lattice during hot rolling may take place as the Ti^4+^ radius (0.64 Å) is close to that of Fe^3+^ (0.68 Å) [28]. On the other hand, the ionic radius of Fe^2+^ (0.78 Å) and Ti^4+^ (0.64 Å) have great differentials, so it is not easy for Ti^4+^ to pass into the crystal lattice of Fe_3_O_4_.

There are three kinds of iron oxides covered on the surface of steel strips. The outermost layer is Fe_2_O_3_ which has a loose structure; the innermost layer is FeO which is denser, and the interlayer is Fe_3_O_4_ which is a mixture of Fe_2_O_3_ and FeO. Though there might be some dissociative Ti^4+^ which can pass into the surface, this would exist in Fe_2_O_3_, which is a porous oxide scale covered on the steel surface. The TiO_2_ nanoparticles would adsorb on the hole of the oxide scale; as a consequence, there is only a chemical adhesion film created on the oxide scales, but no new phase appeared in the metal base.

## 4. Conclusions

The present work analyzed the surface morphology and metallographic structure of hot rolled SS41 steel, lubricated with nano-TiO_2_ lubricating fluid. The following conclusions were drawn from this study:

(1) Adding nano-TiO_2_ in lubricating fluid was associated with a 62.5% increase in the maximum non-seizure load, a 33.8% reduction in the friction coefficient and a 47.4% reduction in the wear extent, compared with traditional lubricating fluid. Moreover, the friction coefficient-time curve of nano-TiO_2_ lubricating fluid became smooth in only 50 s, while that of traditional lubricating fluid presented a remarkably increasing trend till 900 s.
(2) The grain size of rolled steel strips lubricated with nano-TiO_2_ lubricating fluid decreased from 18.51 μm to 7.46 μm. Additionally, the phase composition of the surface still appeared as a mixture of ferrite and pearlite. There was no iron micro-alloyed phenomenon with titanium. The homogeneity of the metal base was not destroyed.
(3) TiO_2_ nanoparticles could deposit on worn surfaces in the hot rolling process. With the generation of iron oxides, nano-TiO_2_ was distributed in the oxide scales diffusely. The compactness of the oxide scales could be drastically enhanced, which caused a slightly increase in Rockwell hardness and a decrease in the thickness of the oxide scales, from 60 μm to 20 μm. There were fewer surface defects on the hot rolled surface.

## Figures and Tables

**Table 1:** Main additives of the nano-TiO_2_ lubricating fluid.

Additive	Content (%)
anatase nano-TiO_2_	0.50–1.50
Glycerin	4.00–8.00
triethanolamine (TEA)	2.00–5.00
sodium polyacrylate (PAAS)	0.02–0.08
sodium hexametaphosphate (SHMP)	0.20–0.60
sodium dodecyl benzene sulfonate (SDBS)	0.50–1.00

**Table 2:** The chemical compositions of the steel strips.

Element	C	Si	Mn	S	P	Cr	Ni	Cu
Content (wt %)	0.20	0.35	1.40	0.045	0.045	0.030	0.030	0.030

**Table 3:** Tribology performances of traditional lubricating fluid and nano-TiO_2_ fluid.

Condition	P_B_/N	Friction Coefficient	Wear Scar Diameter/mm			
			1#	2#	3#	Mean Value
traditional lubricating fluid	392	0.1519	1.182	1.179	1.138	1.166
nano-TiO_2_ lubricating fluid	637	0.1006	0.645	0.580	0.613	0.613

**Table 4:** The average diameters of the grains.

Condition	Average Diameters/μm
non-lubricant	18.51
traditional lubricating fluid	13.86
nano-TiO_2_ lubricating fluid	7.46

**Table 5:** The finishing thicknesses and spring-backs of the hot rolled steel strips.

Condition	Finishing Thickness/mm	Spring-Back/%	Overall Reduction/%
non-lubricant	4.98	24.50	83.4
traditional lubricating fluid	4.57	14.25	84.8
nano-TiO_2_ lubricating fluid	4.13	3.25	86.2

## References

[1] Wu H., Zhao J.W., Xia W.Z., Cheng X.W., He A.S., Jung H.Y., Wang L.Z., Huang H., Jiao S.H., Huang L. (2017). A study of the tribological behaviour of TiO_2_ nano-additive water-based lubricants. Tribol. Int..

[2] Hsu S.M. (2004). Nano-lubrication: Concept and design. Tribol. Int..

[3] Xie H.M., Jiang B., Hu X.Y., Peng C., Guo H.L., Pan F.S. (2017). Synergistic effect of MoS_2_ and SiO_2_ nanoparticles as lubricant additives for magnesium alloy–steel contacts. Nanomaterials.

[4] Liu Y.R., Hu K.H., Hu E.Z., Guo J.H., Han C.L., Hu X.G. (2017). Double hollow MoS_2_ nano-spheres: Synthesis, tribological properties, and functional conversion from lubrication to photocatalysis. Appl. Surf. Sci..

[5] Sayuti M., Sarhan A.D.D., Salem F. (2014). Novel uses of SiO_2_ nano-lubrication system in hard turning process of hardened steel AISI4140 for less tool wear, surface roughness and oil consumption. J. Clean. Prod..

[6] Hu K.H., Xu Y., Hu E.Z., Guo J.H., Hu X.G. (2016). Rolling friction performance and functional conversion from lubrication to photocatalysis of hollow spherical nano-MoS_2_/nano-TiO_2_. Tribol. Int..

[7] Xia W.Z., Zhao J.W., Wu H., Jiao S.H., Jiang Z.Y. (2017). Effects of oil-in-water based nanolubricant containing TiO_2_ nanoparticles on the tribological behaviour of oxidized high-speed steel. Tribol. Int..

[8] Luo T., Wei X.W., Zhao H.Y., Cai G.Y., Zheng X.Y. (2014). Tribology properties of Al_2_O_3_/TiO_2_ nanocomposites as lubricant additives. Ceram. Int..

[9] Totka B., Lucie S., Petra R., Karolína B., Lukas V., Petr L. (2017). The application potential of SiO_2_, TiO_2_ or Ag nanoparticles as fillers in machining process fluids. J. Clean. Prod..

[10] Sun J.L., Zhu Z.X., Xu P.F. (2015). Study on the lubricating performance of nano-TiO_2_ in water-based cold rolling fluid. Mater. Sci. Forum.

[11] Zhu Z.X., Sun J.L., Niu T.L., Liu N.N. (2015). Experimental research on tribological performance of water-based rolling liquid containing nano-TiO_2_. Proc. Inst. Mech. Eng. Part N J. Nanoeng. Nanosyst..

[12] Kumar A.S., Kumar A.T., Kumar R.S., Rai A.D. (2015). Tribological investigation of TiO_2_ nanoparticle based cutting fluid in machining under minimum quantity lubrication (MQL). Mater. Today Proc..

[13] Najiha M.S., Rahman M.M., Kadirgama K. (2016). Performance of water-based TiO_2_nanofluid during the minimum quantity lubrication machining of aluminium alloy AA6061-T6. J. Clean. Prod..

[14] Chetan, Behera B.C., Ghosh S., Rao P.V. (2016). Application of nanofluids during minimum quantity lubrication: A case study in turning process. Tribol. Int..

[15] Jung S.N., Hoon D.K., Chung H., Lee S.W. (2015). Optimization of environmentally benign micro-drilling process with nanofluid minimum quantity lubrication using response surface methodology and genetic algorithm. J. Clean. Prod..

[16] Xia W.Z., Zhao J.W., Wu H.S., Jiao H., Zhao X.M., Zhang X.M., Jiang Z.Y. (2016). A novel nano-TiO_2_ additive oil-in-water lubricant for hot steel rolling. Mater. Sci. Forum.

[17] Zhu Z.X., Sun J.L., Wei H.R., Niu T.L., Zhu Z.L. (2013). Research on lubrication behaviors of nano-TiO_2_ in water-based hot rolling liquid. Adv. Mater. Res..

[18] Okeke G., Hammond R.B., Antony S.J. (2016). Effects of heat treatment on the atomic structure and surface energy of rutile and anatase TiO_2_ nanoparticles under vacuum and water environments. Chem. Eng. Sci..

[19] Nicholas T.N., Michael K.S., Steven J.H., Linda F.H., Suresh C.P. (2010). A systematic study of the effect of silver on the chelation of formic acid to a titanium precursor and the resulting effect on the anatase to rutile transformation of TiO_2_. J. Phys. Chem..

[20] Eleana K., Vassileios D., Tiverios V., Kyriakos B., Alexis L., Christos K. (2015). Comparative study of phase transition and textural changes upon calcination of two commercial titania samples: A pure anatase and a mixed anatase-rutil. J. Solid State Chem..

[21] Cheng L.W., Weng S.H., Hsueh L.C., Huey J.L., Horng H.K., Moo C.W. (2016). Kinetics of anatase transition to rutile TiO_2_ from titanium dioxide precursor powders synthesized by a sol-gel process. Ceram. Int..

[22] Sajad Z., Reza J.N., Masoud A. (2016). The sources of the micro stress and strain inhomogeneity in dual phase steels. Mater. Sci. Eng. A.

[23] Ingole S., Charanpahari A., Kakade A., Umare S.S., Bhatt D.V., Menghani J. (2013). Tribological behavior of nano TiO_2_ as an additive in base oil. Wear.

[24] Kumara P., Banerjee M.K., Hodgson P., Choudhray A.R. (2017). Warm working characterization of low carbon steel in hot rolling. Mater. Today Proc..

[25] Dwivedi D.K. (2010). Adhesive wear behaviour of cast aluminium–silicon alloys: Overview. Mater. Des..

[26] Zhang C., Wu R., Song C., Zhang Y.F., Li S.L., Tan J.M. (2011). The grain refining and strengthening mechanism of C-Mn steel by CSP. Adv. Mater. Res..

[27] Hong Y.S., Hai T.L., Hui H.L., Hao Z.L., Wen Q.L., Xiao M.Z., Guo D.W. (2014). Effect of hot rolling reduction on microstructure, texture and ductility of strip-cast grain-oriented silicon steel with different solidification structures. Mater. Sci. Eng. A.

[28] Ouyang W.Y., Kuna E., Yepez A., Balu A.M., Romero A.A., Colmenares J.C., Luque R. (2016). Mechanochemical synthesis of TiO_2_nanocompositesas photocatalysts for benzyl alcohol photo-oxidation. Nanomaterials.

